# Integrative analysis of 16S rRNA sequencing and network pharmacology suggests protective effects of Sihuang Zhili Granules against APEC O78 challenge through gut homeostasis-related changes

**DOI:** 10.3389/fvets.2026.1789084

**Published:** 2026-05-05

**Authors:** Zikai Guo, Xiaoran Wang, Danwei Cai, Zhennan Guo, Pi Cheng, Jianguo Zeng, Hua Liu, Yisong Liu

**Affiliations:** 1 Hunan Provincial Key Laboratory of Traditional Chinese Veterinary Medicine, College of Veterinary Medicine, Hunan Agricultural University, Changsha, China; 2 Chinese Medicinal Materials Breeding Innovation Center, Yuelushan Laboratory, Changsha, China

**Keywords:** anti-inflammatory, antimicrobial resistance, avian pathogenic *Escherichia coli*, gut microbiota, intestinal barrier, Sihuang Zhili Granules

## Abstract

**Introduction:**

Avian pathogenic *Escherichia coli* (APEC) infection poses a serious threat to the poultry industry and is increasingly difficult to control because of antimicrobial resistance.

**Methods:**

This study combined microbiota analysis and network pharmacology to investigate the protective effects of Sihuang Zhili Granules (SH) against APEC O78 challenge.

**Results:**

Phenotypic analyses showed that SH treatment reduced mortality, partially improved growth-related performance, and alleviated histopathological lesions in the heart, liver, and spleen. At the mechanistic level, SH treatment was associated with increased mRNA expression of intestinal tight junction-related genes, including *Occludin* and *Claudin-3*, reduced serum levels of pro-inflammatory cytokines such as IL-1β, IL-6, and TNF-α, and improvement in selected serum biochemical disturbances induced by APEC O78 challenge. 16S rRNA gene sequencing showed that SH treatment was associated with changes in cecal microbiota composition, including a lower relative abundance of *Escherichia–Shigella* and enrichment of taxa such as members of the Ruminococcaceae family. Integrative network pharmacology and molecular docking analyses further suggested that compounds such as eupatorin and oroxylin A may participate in host responses to APEC infection through candidate inflammation- and barrier-related pathways.

**Discussion:**

These findings support the potential of SH as a candidate intervention for APEC O78-associated disease in broilers, while the precise microbiota-related and molecular mechanisms require further experimental validation.

## 1 Introduction

Bacterial infections represent one of the primary causes of economic losses in the poultry industry (1). Avian pathogenic *Escherichia coli* (APEC), as a highly invasive Gram-negative bacterium, not only causes severe airsacculitis and pericarditis but also leads to perihepatitis and septicemia (2). Currently, the treatment of this disease relies mainly on antibiotics and chemical antimicrobial agents (3). However, their application is limited by issues such as toxic side effects, allergic reactions, superinfection, and bacterial resistance (4). Furthermore, drug residues in animal-derived food products pose a significant threat to human health (5). According to the China Antimicrobial Resistance Surveillance System (CARSS), *Escherichia coli* has consistently ranked among the most frequently isolated Gram-negative pathogens in national surveillance reports (6). More critically, the overuse of antibiotics has exacerbated the problem of antimicrobial resistance, rendering treatments increasingly difficult. In contrast, most single herbal medicines possess a narrow antibacterial spectrum, making them difficult to cope with complex and variable pathological conditions (7). Conversely, compound Traditional Chinese Medicine (TCM) formulas are characterized by multi-components and multi-targets, exhibiting advantages such as a broad antibacterial spectrum, high efficiency, low toxicity, and a lower propensity for inducing drug resistance (8). Therefore, the research and development of green and efficient antibiotic alternatives is urgent (9).

Current research indicates that the core pathogenesis of APEC infection revolves around a classic pathological cascade: intestinal barrier impairment—microbiota dysbiosis—systemic inflammation (10). The intestinal mucosa serves as the primary barrier defending the host against pathogen invasion. In particular, tight junction (TJ) proteins, such as *Occludin* and the *Claudin* family, play a pivotal role in maintaining the integrity of the mechanical barrier (11, 12). Following intestinal colonization, APEC can induce villus atrophy, crypt damage, and the downregulation of TJ. These pathological changes lead to increased mucosal permeability, facilitating the transepithelial translocation of bacteria and their endotoxins (13). This process subsequently triggers acute inflammatory responses and disrupts host immune homeostasis (14).

Characterized by multi-component and multi-target synergism, TCM exhibits unique advantages in combating drug-resistant bacterial infections. This is largely achieved through host-directed therapies, which modulate host immunity and barrier functions rather than relying solely on direct bactericidal activity (15). Sihuang Zhili Granules (SH) is a classic herbal formula composed of *Scutellaria baicalensis* (Huangqin), *Phellodendron amurense* (Huangbai), *Coptis chinensis* (Huanglian), *Rheum palmatum* (Dahuang), and *Isatis indigotica* (Banlangen). Previous studies in poultry have suggested beneficial effects of Sihuang Zhili Granules or related Sihuang-based formulations on diarrhea-related symptoms, immunity, and *Escherichia coli*-associated disease parameters (16, 17). However, despite its remarkable clinical efficacy, the precise pharmacological mechanisms and the core bioactive material basis of SH remain to be systematically elucidated.

Therefore, this study employed multidimensional *in vivo* experiments combined with network pharmacology to explore the protective effects of SH on intestinal morphology, barrier-related parameters, and gut microbiota-associated changes. By focusing on the pathological cascade of intestinal barrier impairment, microbiota dysbiosis, and systemic inflammation, we aimed to identify candidate mechanisms by which SH may influence host responses during APEC infection. These findings may provide experimental support for further studies on SH-based interventions against avian APEC infection.

## 2 Materials and methods

### 2.1 Experimental materials

One-day-old white-feathered broiler chicks (*Gallus gallus*) were purchased from Henan Fengyuan Poultry Co., Ltd. The avian pathogenic *Escherichia coli* strain CVCC1490 (serotype O78) was kindly provided by the College of Animal Science, Hunan Agricultural University.

#### 2.1.1 Sihuang Zhili Granules (SH): source, formulation, and administration

Sihuang Zhili Granules (SH) used in this study were commercially purchased from Baoding Jizhong Pharmaceutical Co., Ltd. (No. 3715, Changcheng South Street, Qingyuan District, Baoding, Hebei, China; veterinary drug approval No. 030065045; batch No. 20240301). The product was a commercial veterinary granule preparation with a specification of 100 g/bag and was packaged in aluminum foil bags. SH is a traditional veterinary herbal formula composed of *Scutellaria baicalensis*, *Phellodendron amurense*, *Coptis chinensis*, *Rheum palmatum*, and *Isatis indigotica*.

For the treatment experiment, SH was administered by feed supplementation at 2 g/kg feed, starting at 14 days of age and continuing for 7 consecutive days. The dosing regimen was selected according to the product usage practice under the present experimental conditions, with reference to previous poultry studies on Sihuang Zhili Granules and related Sihuang-based formulations (16, 17).

### 2.2 Main reagents and instruments

Commercial ELISA kits for chicken IL-1β, IL-6, IL-10, TNF-α, IgG, and IgM were obtained from Shanghai Enzyme-linked Biotechnology Co., Ltd. (Shanghai, China). Serum biochemical parameters, including total protein (TP), albumin (ALB), total cholesterol (TC), triglycerides (TG), high-density lipoprotein cholesterol (HDL-C), low-density lipoprotein cholesterol (LDL-C), creatinine (CREA-S), uric acid (UA), alanine aminotransferase (ALT), and aspartate aminotransferase (AST), were measured using commercial kits on an automatic biochemical analyzer (Mindray, Shenzhen, China). The main instruments used in this study included a Tecan INFINITE E PLEX multimode microplate reader, a Mindray BS-460 automatic biochemical analyzer, and a microscope from Carl Zeiss Suzhou Co., Ltd.

### 2.3 Bacterial strain activation

The CVCC1490 strain, stored at −80 °C, was streaked onto Luria-Bertani (LB) agar plates and incubated at 37 °C for 12 h. Single colonies were picked and inoculated into LB broth, followed by incubation at 37 °C with shaking at 200 rpm for 12 h. Bacterial cells were harvested by centrifugation, and the concentration was adjusted to 1 × 10^9^ CFU/mL using sterile 0.9% sodium chloride solution.

### 2.4 Establishment of the APEC O78 challenge model and therapeutic trial design

#### 2.4.1 Husbandry and basal diet

All animal procedures were reviewed and approved by the Biomedical Research Ethics Committee of Hunan Agricultural University (approval No. 2025–211) and were conducted in accordance with institutional guidelines for animal welfare and care. This study was reported in accordance with the ARRIVE guidelines (18). Broilers had ad libitum access to feed and water. The basal diet was formulated with reference to the Chicken Feeding Standard (NY/T33-2004) (19). The composition and nutrient levels of the basal diet are presented in Supplementary Table S1.

#### 2.4.2 Determination of the APEC O78 challenge dose

Broilers were intraperitoneally injected with 0.2, 0.4, 0.8, or 1.2 mL of APEC O78 suspension (1 × 10^9^ CFU/mL), and mortality was recorded for 7 days. Clinical signs and gross lesions were also monitored to evaluate the severity of infection. An appropriate challenge dose was determined based on the establishment of a stable subacute infection model, defined as the induction of typical clinical signs and lesions of avian colibacillosis together with a cumulative mortality rate of approximately 50–70% within 7 days.

#### 2.4.3 Therapeutic trial design and animal grouping

One-day-old chicks with no significant difference in initial body weight were randomly assigned to three experimental groups. Each group consisted of 3 replicates, with 15 birds per replicate (*n* = 45 per group). The challenge was performed by intraperitoneal injection once daily for three consecutive days (days 11–13). The APEC O78 group (model group) and the SH group were intraperitoneally injected with 0.8 mL of APEC O78 suspension (1 × 10^9^ CFU/mL), while the CON group (blank control) was injected with an equal volume of sterile liquid culture medium. Drug administration commenced at 14 days of age and continued for 7 days, followed by a 2-week observational rearing period. The total experimental duration was 35 days. The therapeutic trial design was developed with reference to the Technical Guidelines for Clinical Trials of Veterinary Traditional Chinese Medicines and Natural Medicines.

#### 2.4.4 Evaluation of therapeutic efficacy

The therapeutic efficacy of SH against avian colibacillosis was evaluated based on clinical indicators, including mentation, number of deaths, mortality rate, clinical recovery rate, total effective rate, and ineffective rate across all experimental groups. In the present study, therapeutic outcomes were evaluated at the final clinical evaluation at 21 days of age, following a 3-day challenge period and 7 consecutive days of treatment. Each bird was assigned to one of four mutually exclusive outcome categories based on clinical observation. “Clinical recovery” was defined as marked improvement or disappearance of clinical signs. “Effective” was defined as partial improvement of clinical signs, whereas “ineffective” was defined as no obvious improvement or worsening of clinical signs. “Death” referred to birds that died during the experimental period. These clinical outcome categories were used for efficacy evaluation only and did not represent microbiological confirmation of APEC O78 clearance. The detailed calculation formulas and specific evaluation criteria are provided in Supplementary method S2.

#### 2.4.5 Sample collection

On day 21 of the experiment (immediately after the 7-day treatment period), two birds were randomly selected from each replicate for blood collection. Blood samples (5 mL) were collected from the jugular vein, allowed to clot at 37 °C for 1 h in an inclined position, and centrifuged at 3,000 rpm for 10 min to obtain serum. Sampling was performed immediately after treatment to evaluate the direct therapeutic effects of SH rather than delayed recovery effects. The serum was stored at −20 °C for subsequent analysis. Following blood collection, the birds were anesthetized with sodium pentobarbital (50 mg/kg, intraperitoneal injection). Adequate anesthetic depth was confirmed by the absence of pedal withdrawal reflex and corneal reflex before euthanasia. The birds were then euthanized by cervical dislocation. All procedures were performed by trained personnel to minimize animal suffering under the approved institutional animal welfare protocol. Tissue samples, including the heart, liver, spleen and intestine (duodenum, jejunum, and ileum), were collected on day 21 of the experiment for histopathology and organ index determination. Organ indices (%) were calculated as (organ weight/body weight) × 100. A portion of each tissue was fixed in 10% formalin solution for histopathological examination via Hematoxylin and Eosin (H&E) staining. The remaining tissue samples were wrapped in aluminum foil and stored at −80 °C for the analysis of intestinal tight junction gene expression.

### 2.5 Measurement indices and methods

#### 2.5.1 Growth performance

Mortality was recorded daily, and body weights were measured at 14 days of age (pre-treatment baseline), 21 days of age (post-7-day treatment), and 35 days of age (after a 2-week observation period). Feed intake was measured in grams using an electronic balance by recording the amount of feed offered and the residual feed for each replicate daily, and was calculated as feed offered minus feed remaining. Average Daily Gain (ADG), Average Daily Feed Intake (ADFI), and Feed-to-Gain ratio (F/G) were calculated for each group. ADG was calculated as body weight gain divided by the number of days, ADFI as total feed intake divided by the number of days, and F/G as feed intake divided by weight gain. For the overall period (days 14–35), ADFI was calculated as the weighted mean of the stage-specific ADFI values for days 14–21 and days 21–35, and F/G was calculated as overall ADFI divided by overall ADG.

#### 2.5.2 Serum biochemical and immune indices

Serum biochemical parameters, including TP, ALB, ALT, AST, TG, TC, HDL-C, LDL-C, CREA-S, and UA, were measured using commercial kits on an automatic biochemical analyzer according to the manufacturers’ instructions. GLOB and the A/G ratio were obtained from the serum biochemical profile and included in the downstream analysis. Serum levels of IL-1β, IL-6, IL-10, TNF-α, IgG, and IgM were measured using commercial ELISA kits according to the manufacturers’ instructions.

#### 2.5.3 Visceral organ pathology and intestinal morphology

Histopathological sections of the heart, liver, spleen and intestine were fixed in 10% formalin, embedded in paraffin, sectioned continuously at 4–5 μm, and stained with hematoxylin and eosin (H&E) for microscopic examination. For intestinal morphometric analysis, observations were performed under a light microscope at ×100 or ×200 magnification, and well-oriented fields with intact structure and a clearly visible villus–crypt axis were selected. Villus height was measured as the vertical distance from the tip of the villus to the crypt opening, and crypt depth was measured as the vertical distance from the crypt opening to the crypt base. The villus height-to-crypt depth ratio (V/C) was then calculated. To reduce subjective bias, all morphometric measurements were performed in a blinded manner. For each bird, 3 non-overlapping fields were randomly selected from each section, and 10 intact villi with the corresponding crypts were measured per field using ImageJ software.

#### 2.5.4 High-throughput sequencing of cecal contents

Cecal content samples were submitted to Shanghai Majorbio Bio-pharm Technology Co., Ltd. for 16S rRNA gene sequencing. Total microbial DNA was extracted from cecal contents and assessed by 1% agarose gel electrophoresis. The V3–V4 region of the bacterial 16S rRNA gene was amplified using primers 338F (5′-ACTCCTACGGG AGGCAGCAG-3′) and 806R (5′-GGACTACHVGGGTWTCTAAT-3′). Amplicon libraries were sequenced on the Illumina PE300 platform. Raw paired-end reads were quality-filtered using fastp (v0.23.4) and merged using FLASH (v1.2.11). High-quality sequences were clustered into operational taxonomic units (OTUs) at 97% sequence similarity using USEARCH/UPARSE (v11), and chimeric sequences were removed during OTU construction. Taxonomic annotation was performed using the RDP Classifier (v2.11) against the SILVA 138.2 16S rRNA database with a confidence threshold of 70%. Sequences annotated as chloroplasts or mitochondria were removed before downstream analyses. For the present study, only samples from the CON, APEC, and SH groups were included in the downstream analyses. Alpha-diversity at the genus level was primarily assessed using the Sobs index. Rarefaction and coverage curves were examined to assess whether sequencing depth was sufficient. Beta-diversity was evaluated by principal coordinate analysis (PCoA) based on Bray–Curtis distances at the genus level, and group differences were further assessed using Adonis analysis with 999 permutations. Taxonomic composition was summarized at the genus level. Differentially abundant taxa among groups were identified by LEfSe analysis, and taxa with an LDA score > 3.5 were considered discriminative features. To explore associations between microbiota changes and host phenotypes, Spearman correlation analysis was performed between selected genera and host indices, including intestinal barrier-related genes (*ZO-1, Occludin, Claudin-1*, and *Claudin-3*) and serum inflammatory or injury-related markers. Microbiota and host parameters used in the correlation analysis were obtained from the same 18 birds.

#### 2.5.5 qRT-PCR

Total RNA was extracted from jejunal tissues using a commercial RNA extraction kit from Accurate Biology according to the manufacturer’s instructions. RNA concentration and purity were assessed before reverse transcription. cDNA was synthesized using a commercial reverse transcription kit from Accurate Biology (China), and quantitative real-time PCR was performed using a SYBR Green-based qPCR kit from Accurate Biology according to the manufacturer’s protocols. *β*-actin was selected as the reference gene based on its common use in previous chicken intestinal gene-expression studies and its routine use in our laboratory under similar experimental conditions. Relative mRNA expression levels of *ZO-1, Occludin, Claudin-1,* and *Claudin-3* were calculated using the 2^-ΔΔCt method. Amplification specificity was assessed based on melting-curve analysis. Six biological replicates per group were analyzed, and each biological replicate was tested in triplicate as technical replicates. The amplification primers were designed and synthesized by Tsingke Biotechnology Co., Ltd. (Beijing, China), and the primer sequences are listed in Table 1. Because no additional amplification-efficiency comparison experiment or formal reference-gene stability analysis was performed in the present study, the qRT-PCR results should be interpreted as relative expression data under the stated experimental conditions.

**Table 1:** Primer sequences used for qRT-PCR analysis.

*Gene names*	Primers sequence (5′ → 3′)	Accession No.
*β-*actin	F: CACCACAGCCGAGAGAGAAAT	NM_205518.2
	R: TGACCATCAGGGAGTTCATAGC	
*ZO-1*	F: GCCAGCCATCATTCTGACTCCAC	XM_015278975.4
	R: GTACTGAAGGAGCAGGAGGAGGAG	
*Occludin*	F: TACGGCAGCACCTACCTCAA	XM_046904540.1
	R: AGGCAGAGCAGGATGACGAT	
*Claudin-1*	F: GCCACGTCATGGTATGGCAA	NM_001013611.2
	R: CCAGCCAATGAAGAGGGCTG	
*Claudin-3*	F: GCCAAGATCACCATCGTCTC	NM_204202.2
	R: CACCAGCGGGTTGTAGAAAT	

*β*-actin was used as the reference gene.

#### 2.5.6 Statistical analysis

Statistical analyses were performed using IBM SPSS Statistics version 27.0 (IBM Corp., Armonk, NY, USA), and figures were prepared using GraphPad Prism version 10.1.2 (GraphPad Software, San Diego, CA, USA). Continuous variables were first assessed for normality and homogeneity of variance. Variables meeting the assumptions of normality and homogeneity of variance were analyzed by one-way ANOVA followed by Tukey’s multiple-comparison test. Variables with unequal variances were analyzed by Welch’s ANOVA followed by the Games–Howell *post hoc* test. Variables that did not satisfy normality assumptions were analyzed using the Kruskal–Wallis test followed by pairwise Mann–Whitney U tests with Holm correction. Categorical clinical outcomes assessed at 21 days of age were compared among groups using Pearson’s chi-square test. For the analysis of therapeutic outcomes shown in Table 2, individual birds were treated as the experimental units, and the analysis was performed on aggregated frequency data using case weighting in SPSS. All expected cell counts were ≥ 5; therefore, an exact test was not required. A *p* value < 0.05 was considered statistically significant.

**Table 2:** Clinical outcome assessment of SH treatment in broilers challenged with APEC O78.

Group	Clinical recovery	Effective	Ineffective	Death	Total effective rate*
CON (*n* = 45)	45 (100)	0 (0.0)	0 (0.0)	0 (0.0)	45 (100.0)
APEC (*n* = 45)	8 (17.8)	6 (13.3)	16 (35.6)	15 (33.3)	14 (31.1)
SH (*n* = 45)	22 (48.9)	9 (20.0)	6 (13.3)	8 (17.8)	31 (68.9)

Clinical recovery was defined as marked improvement or disappearance of clinical signs at the final clinical evaluation. “Effective” referred to partial improvement of clinical signs, whereas “ineffective” referred to no obvious improvement or worsening of clinical signs. These outcome categories were assigned based on clinical observation only and did not represent microbiological confirmation of APEC O78 clearance. Overall differences in outcome distribution among groups were analyzed using Pearson’s chi-square test (*χ*^2^ = 68.834, *p* < 0.001). All expected cell counts were ≥ 5.

*Total effective rate (%) = (number of birds classified as clinical recovery + number of birds classified as effective) / total number of birds × 100.

### 2.6 Network pharmacology analysis

#### 2.6.1 Screening of active ingredients and target prediction

Active ingredients of Sihuang Zhili Granules (SH) were screened from the Traditional Chinese Medicine Systems Pharmacology Database (TCMSP) using oral bioavailability (OB) ≥ 30% and drug-likeness (DL) ≥ 0.18 as filtering criteria. Candidate targets of the selected compounds were obtained from TCMSP and supplemented using SwissTargetPrediction. Because SwissTargetPrediction is based on human and rodent target prediction models rather than chicken-specific models, the predicted targets were used only as exploratory host-related candidate targets for subsequent network analysis. All target names were standardized using the UniProt database.

#### 2.6.2 Acquisition of disease-related targets

Host response-related targets associated with *Escherichia coli* infection were retrieved from the GeneCards,^1^ OMIM,^2^ and DisGeNET^3^ databases using the keyword “*Escherichia coli*.” Because these databases are primarily human disease- and gene-centered resources, the retrieved targets were regarded as exploratory host-associated candidate targets rather than chicken-specific or bacterial targets. The overlapping targets between SH-related targets and *Escherichia coli* infection-related host targets were used for subsequent network analysis.

#### 2.6.3 Construction of the component-target network

The overlapping SH-related targets and host response-related candidate targets associated with *Escherichia coli* infection were identified using the Venny 2.1 online platform.^4^ These overlapping targets were imported into Cytoscape 3.10.1 to construct a compound-target network. Network topology was analyzed using the CytoHubba (20) plugin, and the top 10 core active components were ranked using the maximal clique centrality (MCC) algorithm.

#### 2.6.4 Construction of the protein–protein interaction (PPI) network

To explore the interactions among the overlapping host-associated candidate targets, these targets were imported into the STRING database^5^ to construct an exploratory PPI network. *Gallus gallus* was selected as the species setting, and the minimum required interaction score was set to 0.700 (high confidence). The resulting network was visualized and analyzed using Cytoscape 3.10.1. In the network visualization, node size and color reflected degree centrality, with larger nodes indicating higher degree values. Candidate hub targets were prioritized from the PPI network using the MCC algorithm in the CytoHubba plugin.

#### 2.6.5 Enrichment analysis

The overlapping candidate targets were imported into the DAVID database^6^ for Gene Ontology (GO) enrichment analysis, including Biological Process (BP), Cellular Component (CC), and Molecular Function (MF), as well as Kyoto Encyclopedia of Genes and Genomes (KEGG) pathway enrichment analysis. *Gallus gallus* was used as the species setting for the enrichment analysis, and the default DAVID background was retained. The enriched GO terms and KEGG pathways were ranked according to enrichment significance and used to identify candidate biological processes and pathways potentially associated with the host response to APEC infection.

#### 2.6.6 Molecular docking analysis

Molecular docking was performed to explore the potential binding patterns between selected active ingredients of SH and key candidate targets. The 3D structures of the compounds were obtained from PubChem and TCMSP, and the protein structures were downloaded from the PDB database. Receptor and ligand preparation was carried out using AutoDockTools, and PyMOL was used to remove water molecules and original ligands from the protein structures. Molecular docking was performed using AutoDock Vina. The docking search space was defined by individually setting a grid box for each receptor according to the corresponding ligand-binding region. Binding energy heatmaps were plotted using Origin 2021. A more negative binding energy indicates a stronger predicted binding affinity in silico. Docking poses were visualized using Discovery Studio 2019. No re-docking validation was performed in the present study.

## 3 Results

### 3.1 Dose selection for establishing the APEC O78-infected broilers model

To determine an appropriate challenge dose, broilers were intraperitoneally injected with 0.2, 0.4, 0.8, or 1.2 mL of an APEC O78 suspension (1 × 10^9^ CFU/mL). All challenged birds developed characteristic clinical signs, including depression, ruffled feathers, huddling, and diarrhea, within 8–12 h. Postmortem examination revealed lesions consistent with avian colibacillosis, such as hepatomegaly and hemorrhagic intestinal mucosa. The 7-day cumulative mortality rates were 20% (3/15), 40% (6/15), 53.3% (8/15), and 86.7% (13/15) in the 0.2, 0.4, 0.8, and 1.2 mL groups, respectively. In addition to mortality, clinical signs and their duration were continuously monitored throughout the observation period and were considered when determining the optimal challenge dose. Based on these predefined criteria, 0.8 mL was selected as the challenge dose for subsequent experiments.

### 3.2 Effects of SH on growth performance in APEC O78-infected broilers

As indicated in Table 3, APEC O78 challenge severely compromised growth performance in broilers. During the acute infection phase (days 14–21), body weight gain and ADG were markedly reduced in the APEC group compared with the CON group. SH treatment partially restored these parameters, and both body weight gain and ADG were significantly higher in the SH group than in the APEC group, although they remained significantly lower than those in the CON group. No significant differences in ADFI or F/G were observed among the three groups during days 14–21.

**Table 3:** Effect of SH on growth performance of APEC infected broilers.

Parameter	CON	APEC	SH	*p* value
14 d body weight, g	195.55 ± 3.85	188.89 ± 1.92	196.67 ± 5.77	0.121
21 d body weight, g	419.29 ± 7.25^a^	194.06 ± 47.37^c^	279.15 ± 10.82^b^	< 0.001
35 d body weight, g	1192.50 ± 36.92^a^	628.78 ± 107.68^c^	997.47 ± 47.40^b^	< 0.001
Body weight gain, 14–21 d, g	223.73 ± 7.48^a^	5.17 ± 49.23^c^	82.48 ± 9.09^b^	< 0.001
ADG, 14–21 d, g/d	31.96 ± 1.07^a^	0.74 ± 7.03^c^	11.78 ± 1.30^b^	< 0.001
ADFI, 14–21 d, g/d	45.21 ± 0.17	24.45 ± 14.21	21.60 ± 12.34	0.072
F/G, 14–21 d	1.42 ± 0.04	−2.68 ± 6.83	1.81 ± 0.91	0.373
Body weight gain, 21–35 d, g	773.21 ± 31.85^a^	434.72 ± 118.68^b^	718.32 ± 43.53^a^	0.003
ADG, 21–35 d, g/d	55.23 ± 2.28^a^	31.05 ± 8.48^b^	51.31 ± 3.11^a^	0.003
ADFI, 21–35 d, g/d	49.30 ± 3.77	49.47 ± 17.18	59.38 ± 9.27	0.509
F/G, 21–35 d	0.90 ± 0.09	1.77 ± 0.94	1.15 ± 0.12	0.218
Body weight gain, 14–35 d, g	996.95 ± 33.92a	439.89 ± 108.12^c^	800.80 ± 41.66^b^	< 0.001
ADG, 14–35 d, g/d	47.47 ± 1.62^a^	20.95 ± 5.15^c^	38.13 ± 1.98^b^	< 0.001
ADFI, 14–35 d, g/d	47.94 ± 2.46	41.13 ± 13.30	46.79 ± 9.62	0.667
F/G, 14–35 d	1.01 ± 0.07	2.11 ± 0.98	1.22 ± 0.20	0.123

Values are presented as mean ± SD (*n* = 3 replicates/group). Data were analyzed by one-way ANOVA followed by Tukey’s multiple-comparison test. Different lowercase letters within the same row indicate significant differences among groups (*p* < 0.05). For the overall period (days 14–35), ADFI was recalculated as the weighted mean of the 14–21 d and 21–35 d values, and F/G was recalculated as ADFI/ADG.

During the post-treatment phase (days 21–35), body weight gain and ADG in the SH group showed no significant difference compared with those in the CON group, and both were significantly higher than those in the APEC group, indicating improved growth recovery after SH treatment. No significant differences in ADFI or F/G were observed among the three groups during days 21–35.

Over the overall period (days 14–35), body weight gain and ADG in the SH group remained significantly higher than those in the APEC group but significantly lower than those in the CON group, suggesting partial but incomplete recovery. No significant differences in ADFI or F/G were observed among the three groups during days 14–35.

### 3.3 Therapeutic effects of SH in APEC O78-infected broilers

As shown in Table 2, all birds in the CON group remained healthy throughout the experiment. Consistent with the improvement in growth-related performance observed in Table 3, the SH-treated group also showed an improved clinical outcome profile at the final evaluation on day 21 of age. In addition to the quantitative efficacy classification, challenged broilers in the APEC group generally exhibited poorer clinical condition, including depression, ruffled feathers and huddling, whereas these signs were alleviated to varying degrees in the SH-treated group during the observation period. In contrast, the APEC group showed poor clinical outcomes, with a clinical recovery rate of 17.8%, a total effective rate of 31.1%, and a mortality rate of 33.3%. The SH-treated group showed a clinical recovery rate of 48.9%, a total effective rate of 68.9%, and a mortality rate of 17.8%. Overall, the distribution of clinical outcomes differed significantly among groups (Pearson’s chi-square test, χ^2^ = 68.834, *p* < 0.001), indicating that SH treatment was associated with improved clinical outcomes in broilers challenged with APEC O78.

### 3.4 Effects of SH on organ indices and histopathology in APEC O78-infected broilers

**Figure 1:**
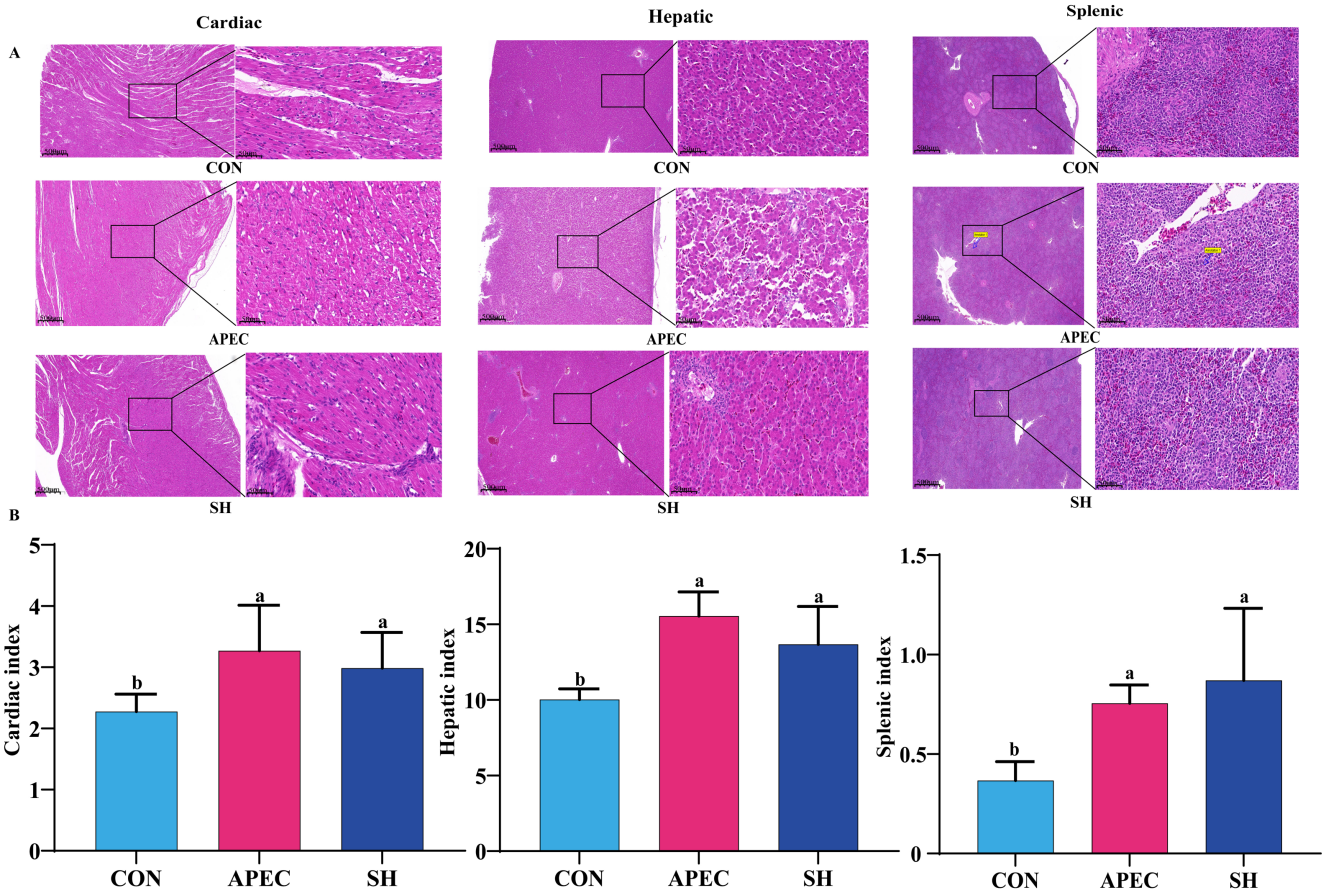
Histopathological sections and organ indices of broilers infected with APEC O78 after SH treatment (day 21). **(A)** Representative H&E-stained sections of cardiac, hepatic, and splenic tissues. **(B)** Quantitative analysis of organ indices. CON, blank control group; APEC, infected model group; SH, treatment group. Data are expressed as mean ± SD (*n* = 6/group). Different lowercase letters indicate significant differences among groups (*p* < 0.05).

To evaluate the systemic impact of the infection and the effects of SH treatment, histopathological changes and organ indices of the heart, liver, and spleen were examined. As shown in Figure 1B, compared to the CON group, the cardiac, hepatic, and splenic indices in the APEC O78 group were significantly elevated (*p <* 0.05), indicating that the infection induced marked organ hypertrophy and edema alongside an inflammatory response. Although the organ indices in the SH treatment group showed a slight downward trend, no statistically significant difference was observed compared to the APEC O78 group. This phenomenon may be attributed to the compensatory hyperplasia of immune organs during the recovery phase.

However, histopathological analysis revealed significant therapeutic benefits (Figure 1A). Organs in the CON group exhibited clear and intact tissue architecture. In contrast, the APEC O78 group displayed severe pathological injuries: the heart showed myocardial fiber disarray and fragmentation; the liver exhibited dissociation of hepatic cords, structural disorder, and obvious inflammatory cell infiltration; and the spleen presented with blurred boundaries between the red and white pulp and lymphocyte depletion. Treatment with SH effectively alleviated these pathological lesions. The SH group exhibited realigned myocardial fibers, restored hepatic lobule architecture with reduced inflammation, and distinct boundaries between the red and white pulp in the spleen.

### 3.5 Effects of SH on intestinal morphology in APEC O78-infected broilers

**Figure 2:**
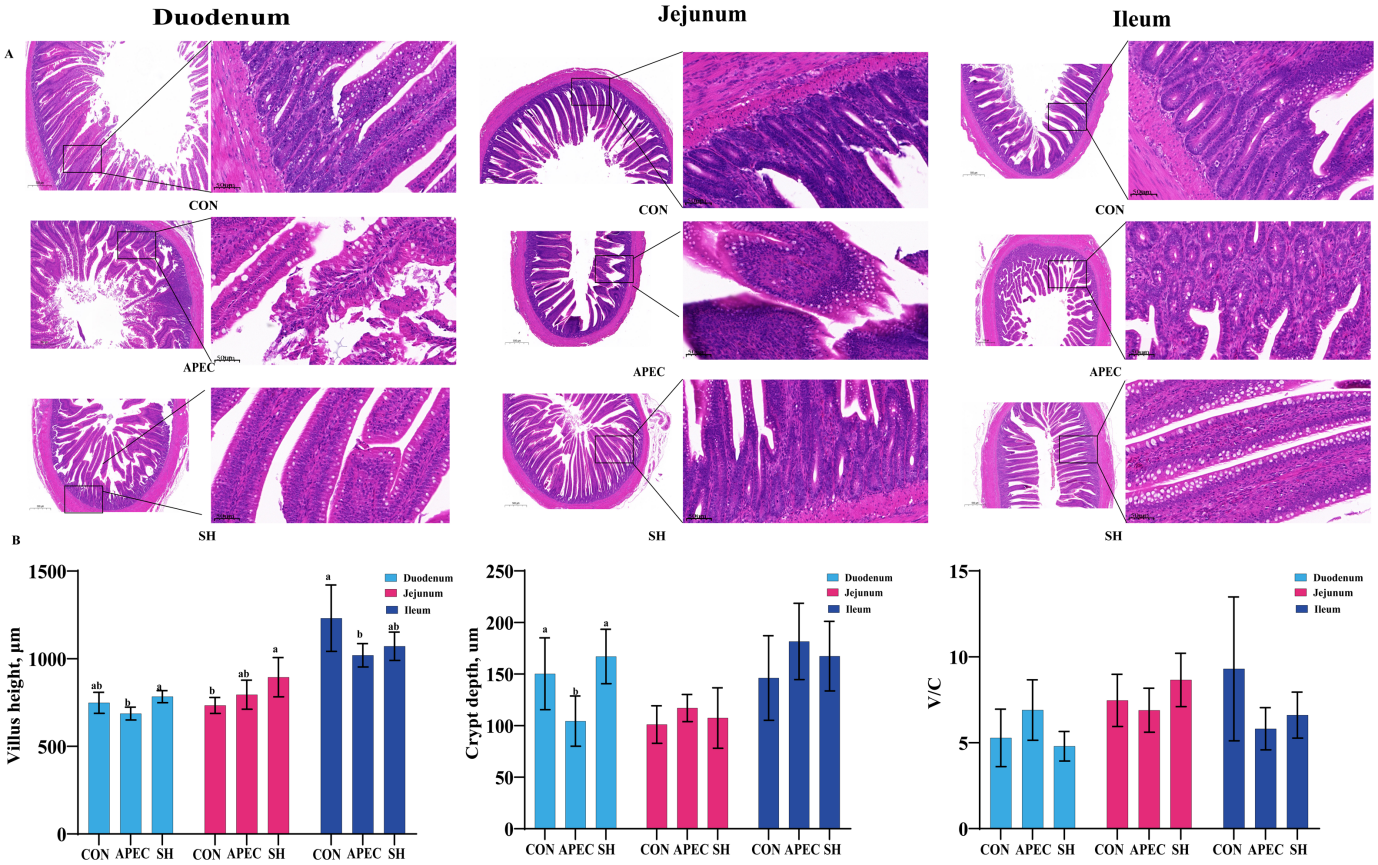
Intestinal histopathological morphology and morphometric analysis in broilers infected with APEC O78 (day 21). **(A)** Representative H&E-stained sections of the duodenum, jejunum, and ileum. **(B)** Quantitative analysis of villus height, crypt depth, and villus-to-crypt ratio (V/C). CON, control group; APEC, infected model group; SH, treatment group. Data are presented as mean ± SD (*n* = 6/group). Variables meeting the assumptions of normality and homogeneity of variance were analyzed by one-way ANOVA followed by Tukey’s multiple-comparison test. Variables with unequal variances were analyzed by Welch’s ANOVA followed by the Games–Howell *post hoc* test. Different lowercase letters indicate significant differences among groups (*p* < 0.05).

Histopathological observations were generally consistent with the intestinal morphometric findings shown in Figure 2. APEC O78 challenge was associated with segment-specific alterations in intestinal morphology. In the duodenum, SH treatment significantly increased villus height and crypt depth compared with the APEC group, indicating amelioration of challenge-associated structural injury. In the jejunum, villus height in the SH group was significantly higher than that in the CON group, whereas the APEC group showed intermediate values. However, no statistically significant differences were observed in duodenal villus-to-crypt ratio (V/C), jejunal crypt depth, jejunal V/C, ileal villus height, ileal crypt depth, or ileal V/C among the groups. Overall, these findings suggest that SH treatment improved selected intestinal morphometric parameters in broilers challenged with APEC O78.

### 3.6 Modulatory effects of SH on the mRNA expression of intestinal tight junction proteins

**Figure 3:**
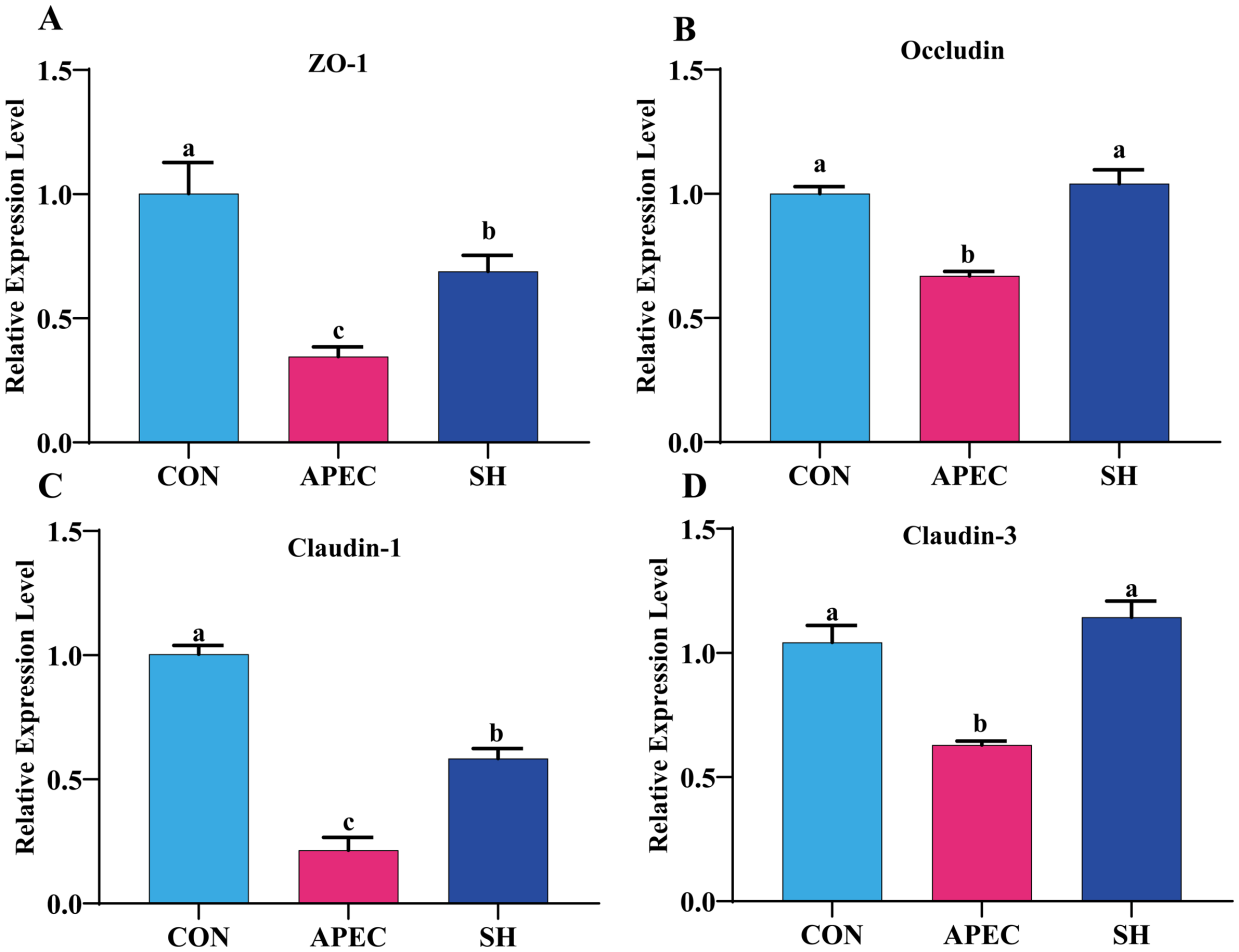
Effects of SH on the mRNA expression of intestinal tight junction-related genes in broilers challenged with APEC O78 (day 21). Relative mRNA expression levels of **(A)** ZO-1, **(B)** *Occludin*, **(C)** *Claudin-1*, and **(D)** *Claudin-3* in jejunal tissues were determined by qRT-PCR. CON, control group; APEC, infected model group; SH, treatment group. Data are presented as mean ± SD (*n* = 6/group). Variables meeting the assumptions of normality and homogeneity of variance were analyzed by one-way ANOVA followed by Tukey’s multiple-comparison test. Variables with unequal variances were analyzed by Welch’s ANOVA followed by the Games–Howell post hoc test. Different lowercase letters indicate significant differences among groups (*p* < 0.05).

To further assess the effect of SH on intestinal barrier-related gene expression, the mRNA levels of key tight junction genes (*ZO-1, Occludin, Claudin-1, and Claudin-3*) in jejunal tissues were measured by qRT-PCR (Figure 3). Compared with the CON group, the APEC group showed significantly lower mRNA expression levels of all four genes (*p* < 0.05). SH treatment markedly increased the expression of *ZO-1, Occludin, Claudin-1*, and *Claudin-3* compared with the APEC group (*p* < 0.05). For all four genes, the expression levels in the SH group were restored to levels statistically comparable to those in the CON group (*p* > 0.05). These findings indicate that SH treatment improved the expression of tight junction-related genes at the mRNA level in broilers challenged with APEC O78.

### 3.7 Effects of SH on serum inflammation and immune indices in APEC O78-infected broilers

To investigate the effects of SH on serum biochemical and immunological alterations induced by APEC O78 challenge, serum biochemical parameters and immune-related factors were evaluated. As shown in Table 4, compared with the CON group, the APEC group exhibited significantly higher levels of ALT, TP, ALB, GLOB, TG, and CREA-S, together with significantly lower HDL-C and TC levels (*p* < 0.05), whereas no statistically significant differences were observed among groups for AST, LDL-C, or UA. After SH treatment, ALT, TP, ALB, GLOB, HDL-C, TC, and CREA-S were significantly improved compared with the APEC group (*p* < 0.05), whereas TG showed an intermediate value and did not differ significantly from either the CON or APEC group. The A/G ratio also differed significantly overall among groups, with the SH group showing a lower value than the CON group, whereas the APEC group was intermediate and did not differ significantly from either group. Overall, these results suggest that SH treatment ameliorated most of the serum biochemical disturbances induced by APEC O78 challenge, although the change in A/G did not follow the same recovery pattern.

**Table 4:** Serum biochemical indicators in broilers challenged with APEC O78.

Items	CON (*n* = 6)	APEC (*n* = 6)	SH (*n* = 6)	*p* value
ALB, g/L	7.34 ± 1.51^a^	11.6 ± 1.95^b^	8.04 ± 1.3^a^	0.003
ALT, U/L	2.30 ± 0.10^b^	5.32 ± 2.42^a^	1.96 ± 0.55^b^	0.005
AST, U/L	156.62 ± 16.44	207.38 ± 76.46	166.88 ± 16.39	0.225
TP, g/L	19.55 ± 4.07^b^	32.84 ± 7.35^a^	23.39 ± 3.04^b^	0.004
HDL-C, mmol/L	1.92 ± 0.35^a^	1.01 ± 0.19^b^	1.62 ± 0.12^a^	<0.001
LDL-C, mmol/L	0.69 ± 0.15	0.51 ± 0.07	0.73 ± 0.28	0.087
TC, mmol/L	1.81 ± 0.35^a^	1.03 ± 0.16^b^	1.55 ± 0.12^a^	<0.001
TG, mmol/L	0.19 ± 0.06^b^	0.28 ± 0.05^a^	0.2 ± 0.04^ab^	0.032
UA, μmol/L	330.08 ± 88.24	453.74 ± 57.23	357.98 ± 74.1	0.052
CREA-S, μmol/L	6.94 ± 1.82^b^	20.40 ± 5.72^a^	8.98 ± 2.45^b^	<0.001
GLOB, g/L	11.41 ± 2.13^b^	21.44 ± 5.2^a^	15.35 ± 1.82^b^	0.002
A/G ratio	0.65 ± 0.09^a^	0.55 ± 0.05^ab^	0.52 ± 0.04^b^	0.021

Values are presented as mean ± SD (*n* = 6/group). The A/G ratio was recalculated for each individual as ALB/GLOB before group statistics. Values with different lowercase letters within the same row indicate significant differences among groups (*p* < 0.05).

**Figure 4:**
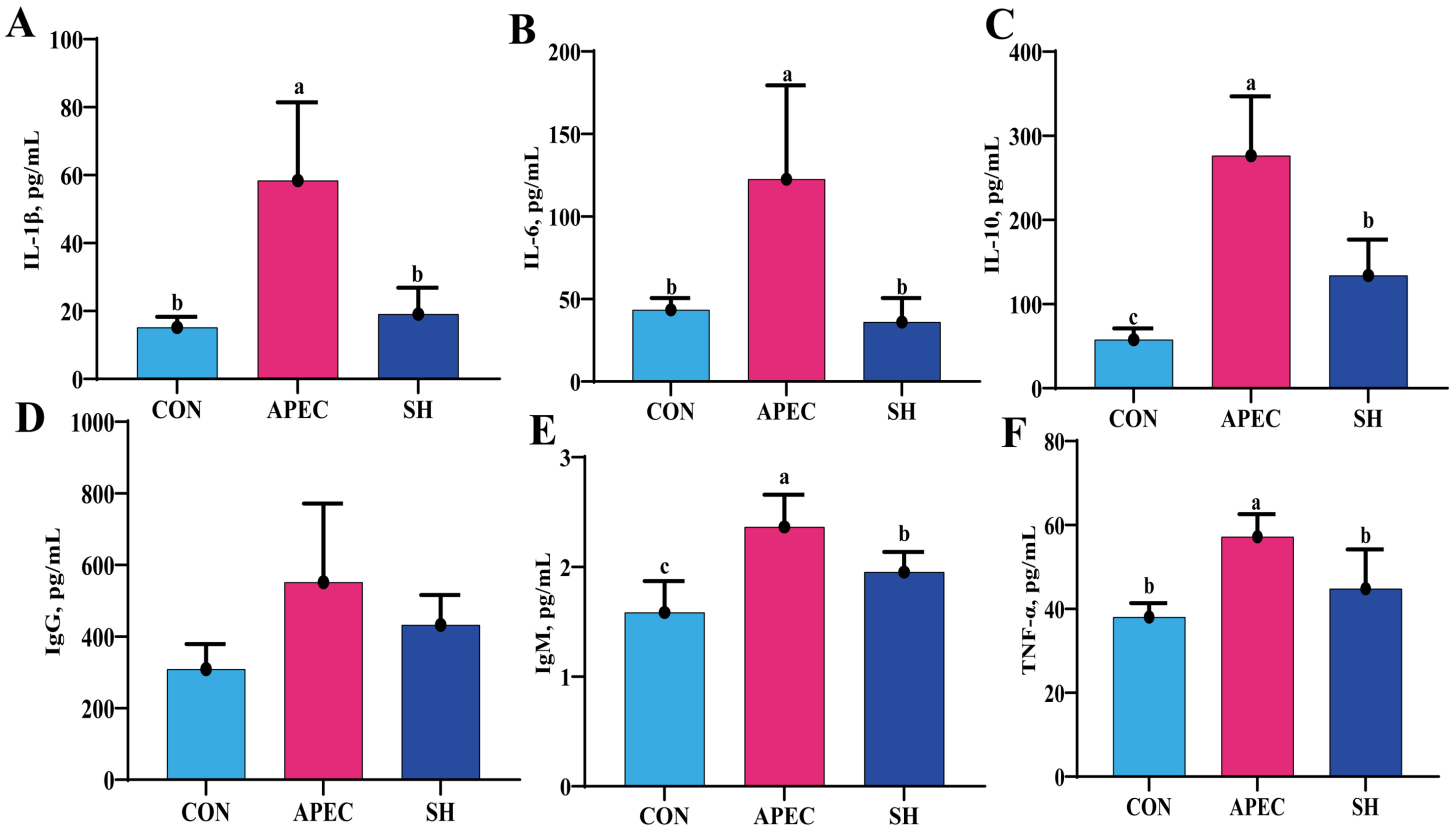
Effects of SH treatment on serum immunological parameters in APEC O78-infected broilers on day 21. **(A)** IL-1β; **(B)** IL-6; **(C)** IL-10; **(D)** IgG; **(E)** IgM; **(F)** TNF-α. CON, control group; APEC, infected model group; SH, treatment group. Data are presented as mean ± SD (*n* = 6/group). Variables meeting the assumptions of normality and homogeneity of variance were analyzed by one-way ANOVA followed by Tukey’s multiple-comparison test. Variables with unequal variances were analyzed by Welch’s ANOVA followed by the Games–Howell post hoc test. Non-normally distributed variables were analyzed using the Kruskal–Wallis test followed by pairwise Mann–Whitney U tests with Holm correction. Different lowercase letters indicate significant differences among groups (*p* < 0.05).

Regarding immune and inflammatory markers (Figure 4), the APEC group showed significantly higher serum levels of IL-1β, IL-6, and TNF-α than the CON group (*p* < 0.05). SH treatment significantly reduced the levels of these pro-inflammatory cytokines compared with the APEC group, and no significant differences were observed between the SH and CON groups (*p* > 0.05). IL-10 was also significantly increased after APEC O78 challenge, and its level in the SH group was intermediate between those of the CON and APEC groups, with significant differences among all three groups (*p* < 0.05). In addition, IgM was elevated in the APEC group compared with the CON group, whereas the SH group showed an intermediate value. No significant difference in IgG was observed among the groups. These findings indicate that SH treatment was associated with attenuation of the excessive inflammatory response induced by APEC O78 challenge and partial modulation of selected immune-related serum indices.

### 3.8 Network pharmacology analysis

**Figure 5:**
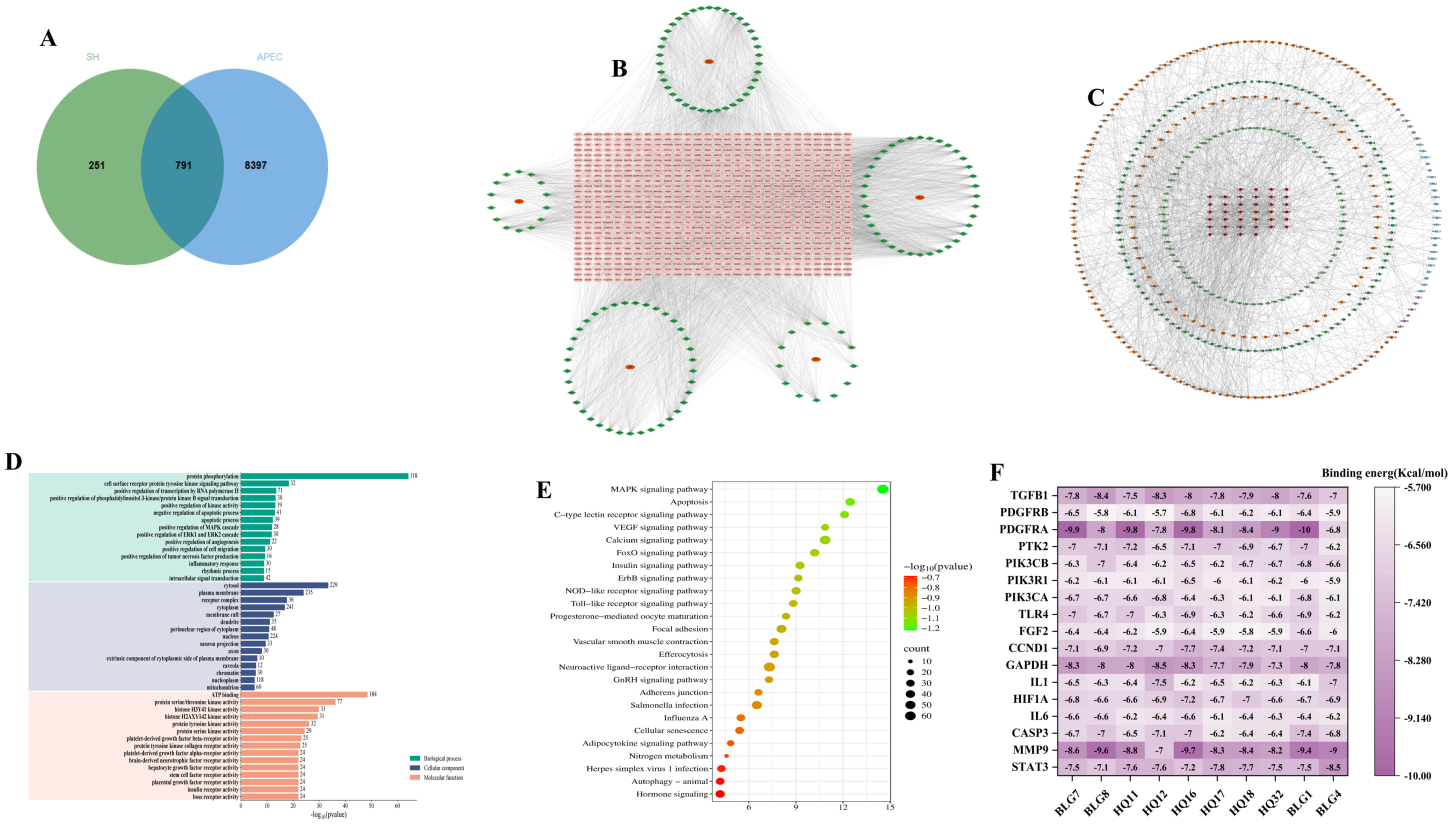
Network pharmacology analysis of SH in the treatment of APEC O78 infection. **(A)** Venn diagram showing the intersection targets between SH active ingredients and APEC-related disease targets. **(B)** Component-target network diagram constructed using Cytoscape. **(C)** Protein–Protein Interaction (PPI) network of the intersection targets. **(D)** Gene Ontology (GO) enrichment analysis of potential targets. **(E)** Kyoto Encyclopedia of Genes and Genomes (KEGG) pathway enrichment analysis. **(F)** Binding Energy heatmap of active ingredients and core targets. SH: Sihuang Zhili Granules; APEC: Avian pathogenic *Escherichia coli*. Enlarged versions of the compound–target network panel **(B)** and the PPI network panel **(C)** are provided in the Supplementary material for improved visualization of the network topology and key nodes.

To explore the multicomponent and multitarget characteristics of SH in relation to host responses during APEC infection, a network pharmacology analysis was performed. First, 1,042 putative targets of the active ingredients in SH were identified from the TCMSP database based on the screening criteria of OB ≥ 30% and DL ≥ 0.18. These were then intersected with exploratory host response-related targets associated with *Escherichia coli* infection retrieved from the GeneCards, OMIM, and DisGeNET databases. A total of 791 overlapping targets were identified (Figure 5A). A compound-target network was then constructed to visualize the multicomponent and multitarget characteristics of SH (Figure 5B). Based on network topology analysis, the top 10 core active components are summarized in Table 5.

**Table 5:** Core active components of SH ranked by the CytoHubba MCC algorithm.

Sequence	Abbreviation	Name	Score
1	BLG4	2-O-beta-D-glucopyranosyl-2H-1,4-benzoxazin-3(4H)-one	88
2	BLG8	3-[[(2R,3R,5R,6S)-3,5-dihydroxy-6-(1H-indol-3-yloxy)-4-oxooxan-2-yl] methoxy]-3-oxopropanoic acid	87
3	HQ12	5,2′,6′-Trihydroxy-7,8-dimethoxyflavone	85
4	HQ11	Salvigenin	85
5	BLG7	Eupatorin	84
6	HQ18	5,7,4′-Trihydroxy-8-methoxyflavone	84
7	HQ16	Oroxylin A	84
8	BLG1	Acacetin	83
9	HQ32	Moslosooflavone	83
10	HQ17	Panicolin	83

The score represents the ranking importance of each component in the component-target network as calculated using the maximal clique centrality (MCC) algorithm in CytoHubba; higher scores indicate greater network centrality.

These overlapping targets were further used to construct a PPI network (Figure 5C). Through network analysis, 17 candidate hub targets were prioritized, including STAT3, MMP9, CASP3, IL6, HIF1A, IL1B, GAPDH, CCND1, FGF2, TLR4, PIK3CA, PIK3R1, PIK3CB, PTK2, PDGFRA, PDGFRB, and TGFB1. GO enrichment analysis suggested that these targets were mainly associated with processes such as regulation of kinase activity, inflammatory response, and apoptosis (Figure 5D). KEGG enrichment analysis showed that these candidate targets were enriched in pathways including MAPK signaling, adherens junction, focal adhesion, Toll-like receptor signaling, and NOD-like receptor signaling (Figure 5E). These enriched pathways may represent candidate mechanisms through which SH influences host responses during APEC infection.

An exploratory molecular docking analysis was subsequently performed between the top 10 core active ingredients and 17 candidate hub targets. As shown in Figure 5F, many compound-target pairs showed negative docking scores, suggesting potential binding interactions in silico. Among the analyzed targets, PDGFRA and MMP9 showed relatively low docking energies with several compounds. Among the compounds, Eupatorin (BLG7), Salvigenin (HQ11), and Oroxylin A (HQ16) showed comparatively strong predicted binding to selected targets. These findings provide in silico prioritization of candidate compound-target pairs for future experimental validation.

#### 3.8.1 Molecular docking analysis of representative compound-target pairs

**Figure 6:**
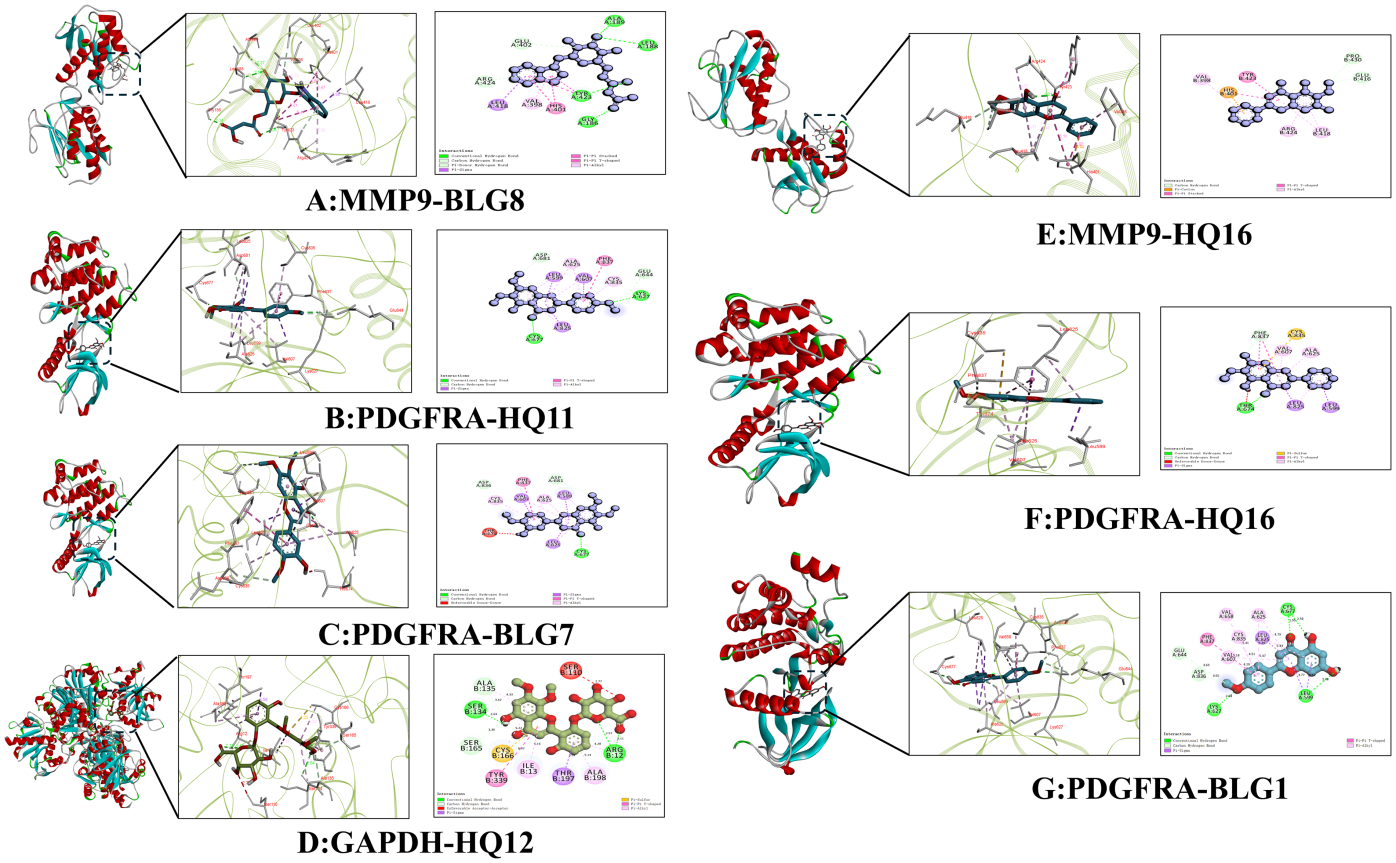
Molecular docking interactions between representative SH active compounds and key target proteins. Representative predicted binding modes of selected compound–target pairs are shown: **(A)** MMP9–BLG8; **(B)** PDGFRA–HQ11; **(C)** PDGFRA–BLG7; **(D)** GAPDH–HQ12; **(E)** MMP9–HQ16; **(F)** PDGFRA–HQ16; and **(G)** PDGFRA–BLG1. Compound abbreviations are defined in Table 5: BLG8, 3-[[(2R,3R,5R,6S)-3,5-dihydroxy-6-(1H-indol-3-yloxy)-4-oxooxan-2-yl] methoxy]-3-oxopropanoic acid; HQ11, Salvigenin; BLG7, Eupatorin; HQ12, 5,2′,6′-Trihydroxy-7,8-dimethoxyflavone; HQ16, Oroxylin A; and BLG1, Acacetin. These docking poses represent in silico structural observations only and should not be interpreted as proof of direct biological modulation.

Representative docking poses of selected compound–target pairs are shown in Figure 6. These representative pairs were selected based on a combination of docking energy, network relevance, and biological interpretability. For MMP9, BLG8 and Oroxylin A (HQ16) showed low docking energies and formed predicted hydrogen-bond interactions with residues such as ARG-424/GLU-402 and TYR-423/GLU-416, respectively. For PDGFRA, Eupatorin (BLG7), Salvigenin (HQ11), and Acacetin (BLG1) also showed predicted binding modes involving residues such as ASP-836, CYS-835, ASP-661, VAL-607, and GLU-644. In addition, HQ12 displayed a predicted binding mode with GAPDH involving residues such as SER-134, THR-197, and ARG-12. These observations provide structural support for the predicted compound–target associations at the docking level, but do not constitute proof of direct biological modulation.

### 3.9 Cecal microbiota composition based on 16S rRNA sequencing

**Figure 7:**
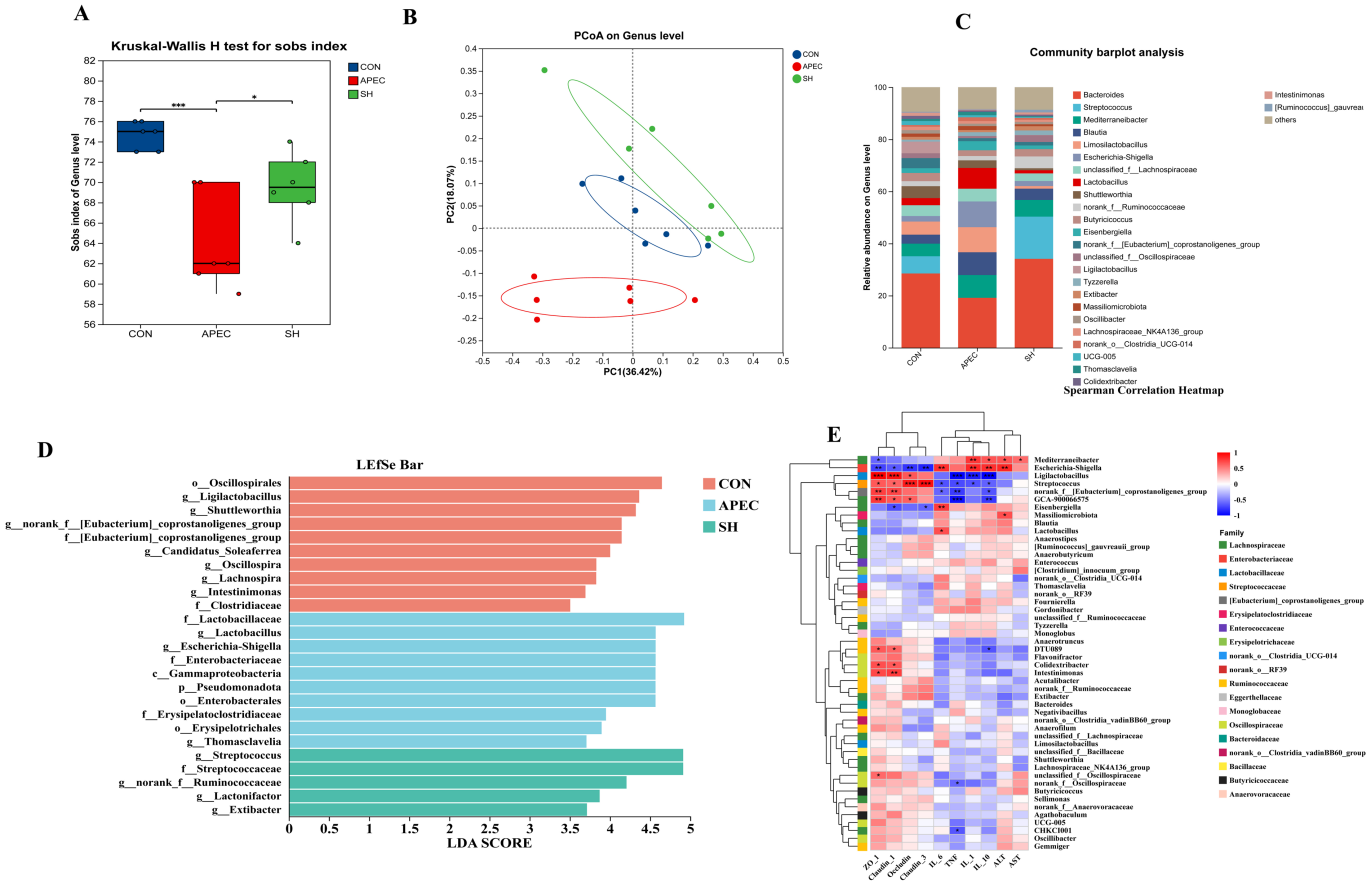
Cecal microbiota alterations associated with SH treatment in broilers challenged with APEC O78. **(A)** Alpha-diversity analysis at the genus level shown by the Sobs index. **(B)** Principal coordinate analysis (PCoA) based on Bray–Curtis distances at the genus level. PC1 and PC2 explained 36.42 and 18.07% of the variation, respectively. **(C)** Relative abundance of the major bacterial genera in each group. **(D)** LEfSe analysis showing discriminative taxa among groups (LDA > 3.5). **(E)** Spearman correlation heatmap between selected bacterial genera and host parameters, including intestinal barrier-related genes and serum inflammatory or injury-related indices. CON, control group; APEC, infected model group; SH, treatment group. Microbiota and host parameters in panel E were obtained from the same 18 birds. Statistical interpretation of panel **(E)** is limited to association analysis and does not imply causality.

To investigate microbiota changes associated with SH treatment in APEC O78-challenged broilers, 16S rRNA gene sequencing was performed on cecal contents from the CON, APEC, and SH groups. A total of 18 samples were included in the present analysis. Rarefaction and coverage analyses (Supplementary Figure S1) indicated that sequencing depth was generally sufficient for downstream community analysis. As shown in Figure 7A, alpha-diversity analysis at the genus level showed that the Sobs index was significantly decreased in the APEC group compared with the CON group, whereas SH treatment increased the Sobs index relative to the APEC group, suggesting that APEC challenge was associated with reduced microbial richness and that SH treatment partially alleviated this change. As shown in Figure 7B, principal coordinate analysis (PCoA) based on Bray–Curtis distances at the genus level showed relative separation among the three groups, with PC1 and PC2 explaining 36.42 and 18.07% of the variation, respectively. Group-level differences in beta-diversity were further supported by Adonis analysis, which showed a significant effect of grouping on the overall microbial community structure at the genus level (*F* = 2.937, R^2^ = 0.281, *p* = 0.003). These results support differences in overall microbial community structure among groups.

As shown in Figure 7C, taxonomic composition analysis at the genus level showed that the APEC group had a higher relative abundance of *Escherichia–Shigella* than the CON group, whereas the SH group showed a lower relative abundance of this taxon than the APEC group. In addition, taxa such as *Bacteroides* were relatively more abundant in the SH group. Because these data are based on genus-level relative abundance profiles, they indicate shifts in microbial composition rather than microbiological clearance of APEC O78. As shown in Figure 7D, LEfSe analysis (LDA > 3.5) identified distinct discriminatory taxa among groups. The APEC group was enriched in taxa related to *Pseudomonadota*, *Gammaproteobacteria*, *Enterobacterales*, *Enterobacteriaceae*, and *Escherichia–Shigella*, whereas the SH group was enriched in taxa including *Lactonifactor*, *Extibacter*, *Streptococcus*, *Thomasclavelia*, and *norank_f__Ruminococcaceae*. These findings support that SH treatment was associated with shifts in selected bacterial taxa in APEC O78-challenged broilers. As shown in Figure 7E, Spearman correlation analysis was performed using microbiota and host parameters obtained from the same 18 birds. The correlation heatmap showed that *Escherichia–Shigella* was positively correlated with serum inflammatory and injury-related indices, including IL-6, TNF-α, ALT, and AST, and negatively correlated with intestinal barrier-related genes such as ZO-1 and *Occludin*. In contrast, taxa enriched in the SH group, including *Ligilactobacillus* and Ruminococcaceae-related genera, tended to show the opposite pattern. These results indicate associations between selected bacterial taxa and host parameters, but they do not establish causal or mechanistic relationships.

## 4 Discussion

Current therapeutic strategies for bacterial infections rely heavily on synthetic antimicrobials, which are often associated with high costs, adverse side effects, and the acceleration of antibiotic-resistant strain emergence (21). Therefore, this study combined *in vivo* evaluation with microbiota analysis, network pharmacology, and molecular docking to explore the protective effects of SH against APEC infection in broilers. Our findings demonstrated that APEC infection markedly impaired growth performance and induced pathological injury in multiple organs. During the acute phase (days 14–21), body weight gain and ADG were markedly reduced after APEC challenge, whereas SH treatment partially restored these parameters. During the recovery phase (days 21–35), body weight gain and ADG in the SH group were restored to levels comparable to those in the CON group and remained significantly higher than those in the APEC group, indicating improved post-treatment growth recovery. This pattern was broadly consistent with the improved final clinical outcome profile observed in the SH-treated group. Over the overall period (days 14–35), growth performance in the SH group remained intermediate between the CON and APEC groups, suggesting partial but incomplete recovery. This phenotypic improvement was accompanied by alleviation of tissue injury in major organs and may be related, at least in part, to the improved intestinal morphology observed in this study, including increased villus height in selected intestinal segments. In addition, SH treatment improved several serum biochemical alterations induced by APEC O78 challenge, including ALT, TP, ALB, GLOB, HDL-C, TC, and CREA-S, suggesting partial alleviation of systemic metabolic and organ-function disturbances associated with infection.

The integrity of the intestinal epithelial barrier is important for limiting bacterial translocation (22). In the present study, APEC challenge was associated with alterations in intestinal morphology and reduced mRNA expression of tight junction-related genes, indicating impairment of intestinal barrier-related parameters (13). SH treatment increased the mRNA expression of ZO-1, Occludin, Claudin-1, and Claudin-3 compared with the APEC group, and for all four genes the SH group reached levels statistically comparable to those of the CON group at the mRNA level. Occludin is an important transmembrane component involved in the regulation of paracellular permeability (11, 12). Therefore, the present data suggest that SH may help maintain intestinal barrier-related status by improving the expression of tight junction-related genes, although direct functional validation of epithelial permeability was not performed in this study.

Gut dysbiosis is widely considered to aggravate intestinal barrier impairment during bacterial infection. In the present study, the APEC O78 group showed an increased relative abundance of *Escherichia–Shigella*, whereas the SH group was enriched in taxa such as *Ruminococcaceae*, which is generally consistent with previous reports linking intestinal dysbiosis to inflammation-associated microbial expansion and loss of beneficial commensals (23, 24). Members of the *Ruminococcaceae* family have been reported to contribute to gut homeostasis through the production of short-chain fatty acids, particularly butyrate (25). In addition, butyrate has been implicated in the regulation of intestinal barrier-related genes, including *Occludin*, in other experimental settings (26). However, because SCFA concentrations were not directly measured in the present study, these results should be interpreted cautiously. This interpretation is also consistent with the observed associations between Ruminococcaceae-related taxa and barrier-related gene expression in the correlation analysis, although the underlying mechanism remains to be verified experimentally (27).

Beyond barrier-related changes, regulation of the host inflammatory response may also contribute importantly to the protective effects of SH against APEC O78 challenge. In the present study, SH treatment significantly reduced the serum levels of IL-1β, IL-6, and TNF-α, indicating attenuation of the excessive systemic inflammatory response induced by infection (28, 29). Together with the observed improvement in intestinal barrier-related parameters and microbiota composition, these findings suggest that the beneficial effects of SH may involve coordinated changes in inflammation-related and gut homeostasis-related parameters.

To further explore the possible compound basis and candidate pathways underlying these effects, network pharmacology combined with molecular docking was performed. The results suggested that compounds such as Eupatorin and Oroxylin A may participate in host responses to APEC infection through candidate inflammation- and barrier-related pathways, including MAPK signaling, adherens junction, and focal adhesion pathways. Eupatorin, a naturally occurring polymethoxyflavone, has been reported to possess antioxidant and anti-inflammatory activities, including suppression of mediators such as NF-κB, COX-2, and IL-6 (30). Oroxylin A, a major flavonoid derived from Scutellaria baicalensis, has also been reported to exert anti-inflammatory and antioxidant effects through pathways including NF-κB/MAPK signaling (31, 32). These previous findings are broadly consistent with the present predictions.

In addition, molecular docking suggested potential binding interactions between selected compounds and inflammation- or repair-related targets, such as PDGFRA and MMP9, thereby providing in silico support for candidate compound-target associations. Taken together, these findings are consistent with the possibility that SH may exert protective effects through combined regulation of inflammatory responses and barrier-related host pathways, while the precise molecular mechanisms remain to be validated experimentally.

Although our network pharmacology analysis suggested the possible involvement of TLR4/NF-κB signaling, this study did not include direct experimental validation at the protein level, which remains a limitation and warrants further investigation.

## 5 Conclusion

In conclusion, SH treatment reduced mortality, partially improved growth performance, alleviated inflammatory responses, improved selected serum biochemical disturbances, and partially restored intestinal barrier-related parameters in broilers challenged with APEC O78. SH treatment was also associated with shifts in cecal microbiota composition, including a lower relative abundance of *Escherichia–Shigella* and enrichment of taxa such as Ruminococcaceae. Integrative analysis further suggested that compounds such as Eupatorin and Oroxylin A may participate in the host response to APEC infection through candidate inflammation- and barrier-related pathways. These findings support the potential of SH as a candidate intervention for APEC O78-associated disease in broilers, while the precise microbiota-mediated and molecular mechanisms require further experimental validation.

## Data Availability

The 16S rRNA gene sequencing data presented in the study are deposited in the NCBI BioProject repository under accession number PRJNA1456045. Additional data supporting the findings of this study are included in the article/Supplementary material, and further inquiries can be directed to the corresponding authors.

## Footnotes

1
https://www.genecards.org/

2
https://omim.org/

3
https://www.disgenet.org/

4
https://bioinfogp.cnb.csic.es/tools/venny/

5
https://string-db.org/

6
https://davidbioinformatics.nih.gov/
