# Supplementary material for: Integrative analysis of 16S rRNA sequencing and network pharmacology suggests protective effects of Sihuang Zhili Granules against APEC O78 challenge through gut homeostasis-related changes
Source: Front Vet Sci. 2026 May 5;13:1789084. doi: 10.3389/fvets.2026.1789084 (PMC13183549; doi:10.3389/fvets.2026.1789084)

Integrative Analysis of 16S rRNA Sequencing and Network Pharmacology Suggests Protective Effects of Sihuang Zhili Granules Against APEC O78 Challenge Through Gut Homeostasis-Related Changes

**Table of contents**

Table S1: Composition and nutrient levels of the basal diet.

Supplementary Method 1: Experimental Diets and Husbandry.

Supplementary Method 2: Evaluation Criteria for Therapeutic Efficacy.

Supplementary Figure S1: Rarefaction and coverage analyses of the cecal 16S rRNA sequencing dataset.

Supplementary Figure S2: Enlarged version of the compound–target network shown in Figure 5B.

Supplementary Figure S3: Enlarged version of the protein–protein interaction (PPI) network shown in Figure 5C.

**Supplementary Table 1. Composition and nutrient levels of the basal diet (air-dry basis)**

| Items | Content | |
| --- | --- | --- |
|  | Days 1–21 | Days 22–35 |
| Ingredients |  |  |
| Corn | 54.265 | 56.266 |
| Soybean meal | 38.259 | 34.82 |
| Soybean oil | 3.051 | 4.986 |
| Dicalcium phosphat | 2.136 | 1.819 |
| Limestone | 0.792 | 0.78 |
| Salt | 0.3 | 0.28 |
| Choline chloride | 0.1 | 0.1 |
| Trace Mineral Premix | 0.2 | 0.2 |
| Lysine | 0.214 | 0.149 |
| Methionine | 0.326 | 0.279 |
| Threonine | 0.07 | 0.03 |
| Valine | 0.047 | 0.021 |
| IU20000 | 0.02 | 0.02 |
| Zeolite powder | 0.05 | 0.05 |
| NSP enzyme | 0.02 | 0.02 |
| Total | 100.0 | 100.0 |
| Nutrient levels |  |  |
| kcal/kg | 2950 | 3100 |
| CP | 22.5 | 21 |
| Ca | 0.96 | 0.87 |
| Available phosphorus | 0.5 | 0.45 |
| Lys | 1.25 | 1.12 |
| Met | 0.635 | 0.571 |
| Val | 0.95 | 0.86 |
| Thr | 0.84 | 0.75 |

Note: The premix provided the following per kilogram of diet: Vitamin A, 9000 IU; Vitamin D3, 3000 IU; Vitamin E, 26 mg; Vitamin K3, 1.20 mg; Vitamin B1, 3.00 mg; Vitamin B2, 8.00 mg; Vitamin B6, 4.40 mg; Vitamin B12, 0.012 mg; Niacin, 45 mg; Folic acid, 0.75 mg; Biotin, 0.20 mg; Calcium pantothenate, 15 mg; Iron (Fe), 100 mg; Copper (Cu), 10 mg; Zinc (Zn), 108 mg; Manganese (Mn), 120 mg; Iodine (I), 1.5 mg; Selenium (Se), 0.35 mg.

Metabolizable energy (ME) values were calculated; other values were measured.

**Supplementary Method 1: Experimental Diets and Husbandry.**

The broiler rearing trial was conducted at the experimental base of the College of Veterinary Medicine, Hunan Agricultural University. The cleaning and disinfection of the poultry house, as well as environmental management, including lighting, temperature, and humidity, were performed according to standard husbandry procedures under the present experimental conditions.

**Supplementary Method 2: Evaluation Criteria for Therapeutic Efficacy**

The therapeutic efficacy was assessed using the following calculations and definitions:

Clinical Recovery: Birds exhibited marked improvement or disappearance of clinical signs at the final clinical evaluation on day 21.

Effective: Birds showed partial improvement of clinical signs at the final clinical evaluation on day 21.

Ineffective: Birds showed no obvious improvement or worsening of clinical signs at the final clinical evaluation on day 21.

Death: Birds that died before the final clinical evaluation were classified as death.

**Supplementary Figure S1.** Rarefaction and coverage analyses of the cecal 16S rRNA sequencing dataset.


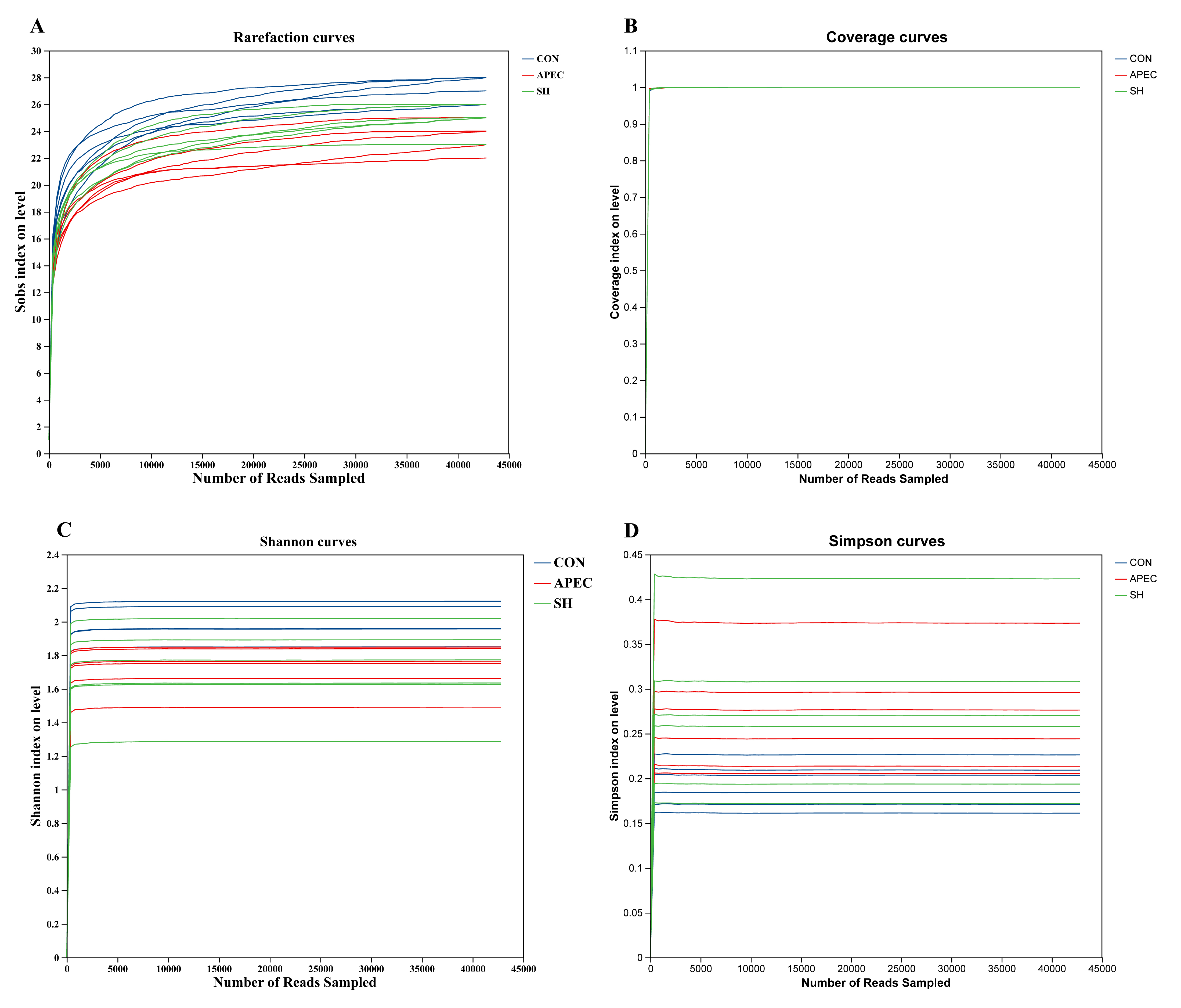


(A) Rarefaction curves based on the Sobs index at the genus level. (B) Coverage curves at the genus level. CON = control group; APEC = infected model group; SH = treatment group. These curves indicate that sequencing depth was generally sufficient for downstream microbial community analysis.

**Supplementary Figure S2**. Enlarged version of the compound–target network shown in Figure 5B.


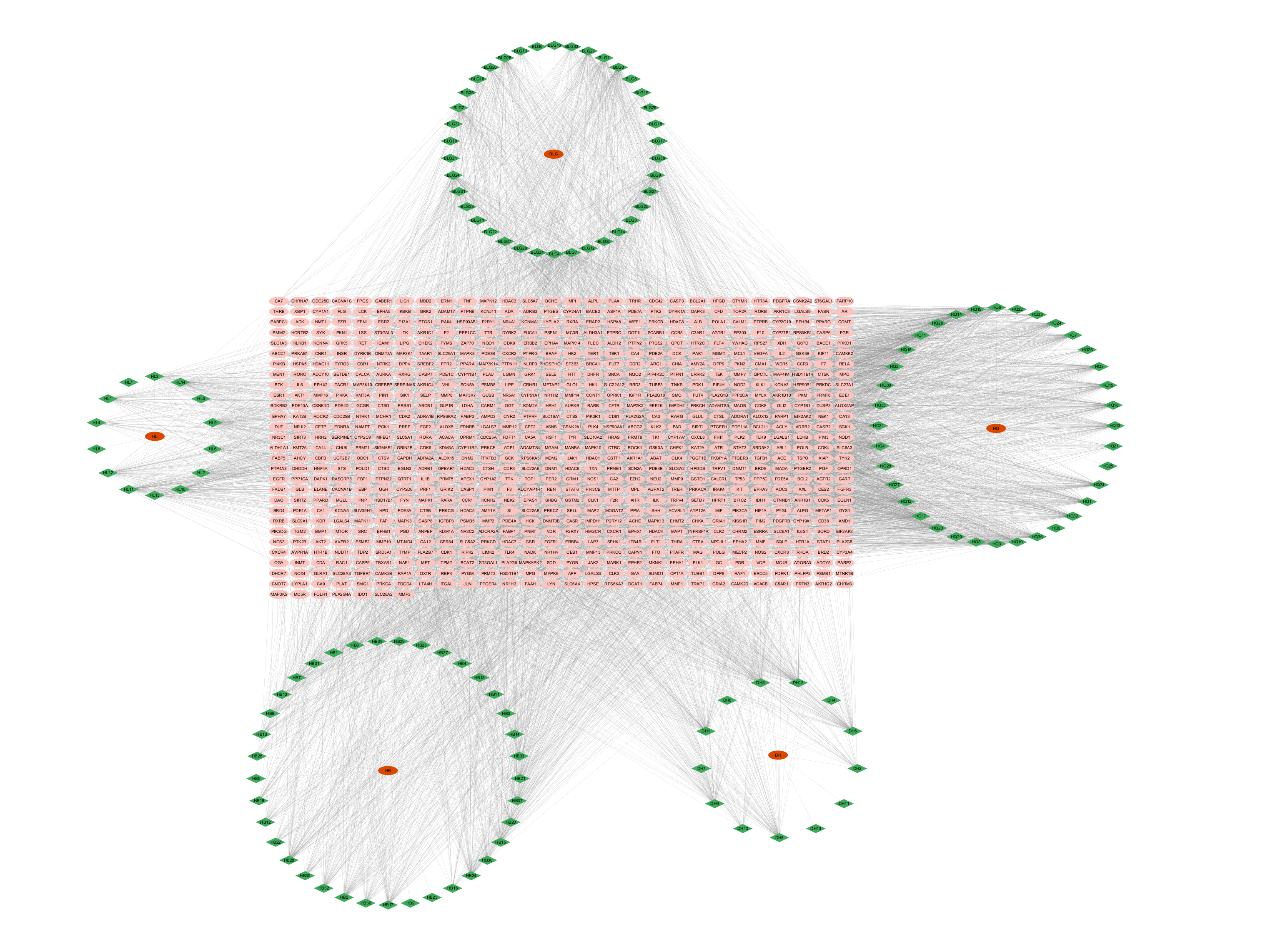


**Supplementary Figure S3**. Enlarged version of the protein–protein interaction (PPI) network shown in Figure 5C.


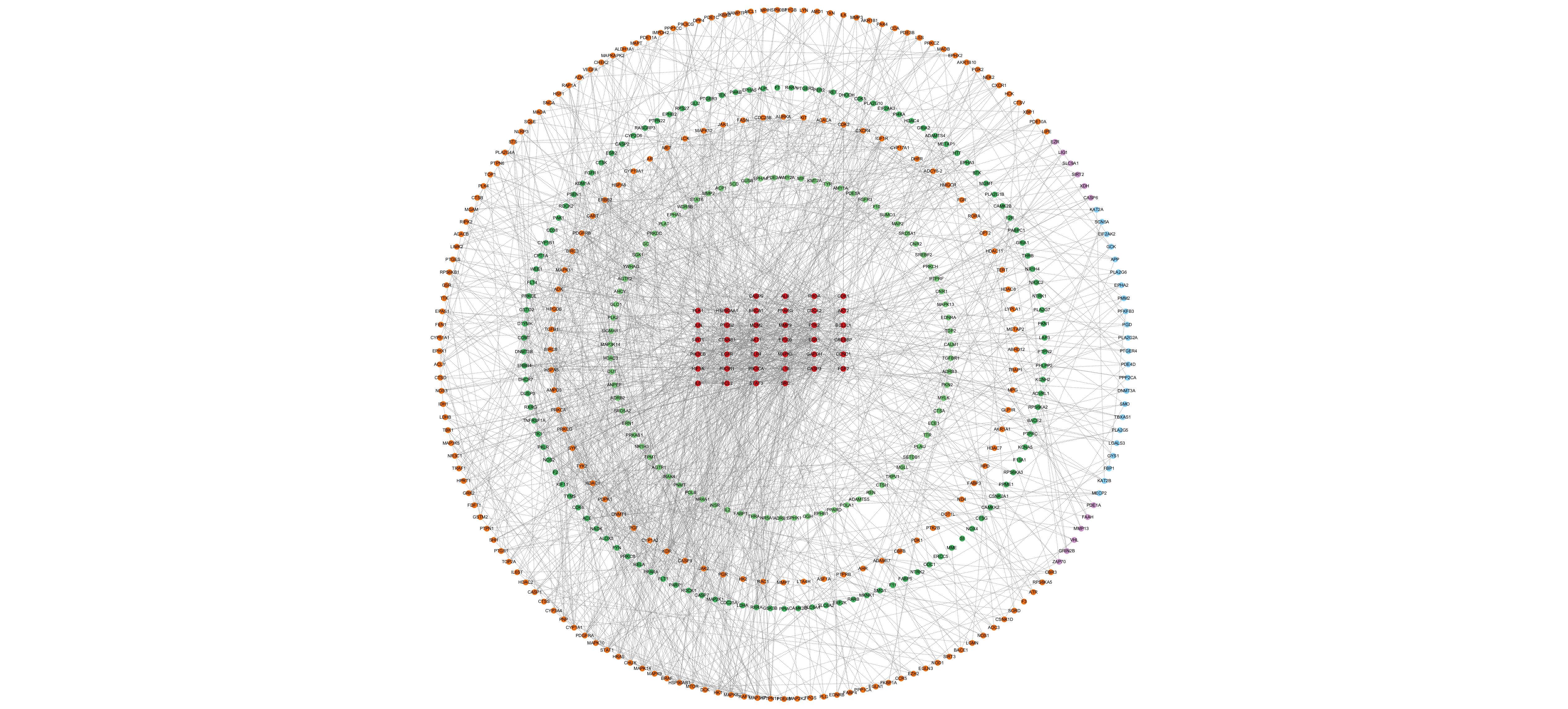

Supplement: Supplementary file 1 [file Data_Sheet_1.docx]
